# Supplementary material for: Reproduction under light pollution: maladaptive response to spatial variation in artificial light in a glow-worm
Source: Proc Biol Sci. 2020 Jul 15;287(1931):20200806. doi: 10.1098/rspb.2020.0806 (PMC7423653; doi:10.1098/rspb.2020.0806)
Supplement: Supplementary material for: Reproduction under light pollution: maladaptive response to spatial variation in artificial light in a glow-worm [file rspb20200806supp1.pdf]

Reproduction under light pollution: maladaptive response to spatial variation in artificial light in a glow-worm

Christina Elgert[1,3], Juhani Hopkins[2,3], Arja Kaitala[2,3], and Ulrika Candolin[1,3]

[1] Organismal and Evolutionary Biology, University of Helsinki, PO Box 65, 00014 Helsinki, Finland

[2] Department of Ecology and Genetics, University of Oulu, PO Box 3000, 90014 Oulu, Finland

[3]Tvärminne Zoological Station, University of Helsinki, J.A. Palméns väg 260, 10900 Hanko, Finland

Doi: 10.1098/rspb.2020.0806

Supplementary Information

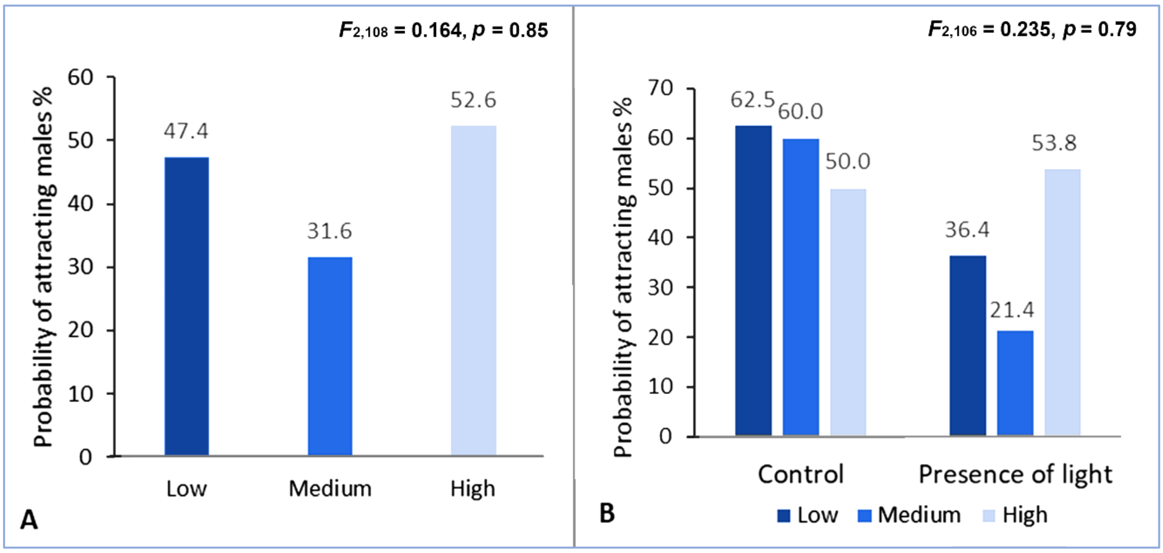

Figure S1: A) The probability that the dummy females with different brightness (low, medium, high) attracted one or more males B) The probability that the dummy females with different brightness (low, medium, high) attracted one or more males in the absence (control) and presence of artificial light from a light pole. Control; low: n = 6; medium: n = 5; high: n = 8. Presence of light: low: n = 13; medium: n = 14; high: n = 11.
